# Prevalence of Retinopathy in Prediabetic Populations: A Systematic Review and Meta-Analysis

**DOI:** 10.7759/cureus.49602

**Published:** 2023-11-28

**Authors:** Manjiri P Sune, Mona Sune, Pradeep Sune, Archana Dhok

**Affiliations:** 1 Ophthalmology, Jawaharlal Nehru Medical College, Datta Meghe Institute of Higher Education and Research, Wardha, IND; 2 Ophthalmology, Sune Eye Hospital, Wardha, IND; 3 Biochemistry, Jawaharlal Nehru Medical College, Datta Meghe Institute of Higher Education and Research, Wardha, IND

**Keywords:** diabetic retinopathy, impaired glucose tolerance, prevalence, impaired fasting glucose, prediabetes

## Abstract

Among the leading causes of vision impairment and blindness globally, diabetic retinopathy (DR) is one of the most important causes. There is increasing evidence of DR prevalence in the prediabetic population. This systematic review presents collective data on retinopathy in the prediabetic population. This review article aimed to estimate the reported prevalence of retinopathy in prediabetes, impaired glucose tolerance test (GTT) without diabetes mellitus, and the risk factors involved and to summarize it. Literature searches were done using the Web of Science, CINAHL, Google Scholar, Cochrane, EMBASE, and PubMed databases from inception to April 2023. Our search included the words prediabetes, DR, and risk factors. All searches were looked at for methodological quality and evidence. Thirty-one studies were included after the screening. Population-based data were used in 23 studies (82.1%). The prediabetic population screened was 10,539. The prevalence of retinopathy ranged between 0.3% and 20.9%, showing a median of 8.1% with an interquartile range (IQR) of 4.2-11%, showing great variance in estimates due to the use of different screening methods, methods used for retinopathy grading, and study populations. Several studies compared the population with normal GTT with impaired glucose tolerance (IGT) and inferred that there was a lower prevalence of retinopathy in the normal GTT population (3.0%, IQR 0.3-7.4%) than prediabetes (6.7%, IQR 1.9-10.1%). According to this data, a greater retinopathy prevalence was found in prediabetic populations.

## Introduction and background

The definition of prediabetic is a higher blood glucose level that does not reach the threshold of type 2 diabetes mellitus [1-4]. All over the world, approximately 374 million people (7.3%) are shown to have prediabetic status, and most of the individuals are unaware of the diagnosis. The International Diabetes Federation predicts that the prevalence of prediabetes will rise to 8.3% among the global adult population by the year 2045. The World Health Organization (WHO) definition of prediabetes is as follows: (a) impaired fasting glucose (IFG) is said when fasting plasma glucose (FPG) is 6.1-6.9 mmol/L (110-125 mg/dL) and (b) impaired glucose tolerance (IGT) is said when two-hour plasma glucose is 7.8-11.0 mmol/L (140-200 mg/dL) after ingestion of 75 g of oral glucose or a combination of the two based on a two-hour oral glucose tolerance test (OGTT) [1]. The cut-off value for IGT used by the American Diabetes Association (ADA) is 140-200 mg/dL, and for IFG, it is 100-125 mg/dL, which is a lower cut-off value than IGT [4].

ADA included hemoglobin A1c (HbA1c) levels ranging from 5.7% to 6.4% along with the above criteria to define the prediabetic state. Current diagnostic levels for fasting and two-hour post-glucose load plasma glucose levels are based on the estimation of the presence of diabetic retinopathy (DR) in the literature available [5]. Several studies have evaluated the prevalence of prediabetes worldwide: WHO criteria in the ESTEBAN French survey and in an English national cohort state that the prevalence of DR was 9.9% and 11%, respectively, and according to ADA criteria in Luxembourg and South Korea, it was 25% and 23.9%, respectively [6]. The population with prediabetes has shown a greater risk of converting to type 2 diabetes mellitus in the future [7]. Every year, around 5-10% of people with prediabetes become diabetic, and according to current data, up to 70% of prediabetic states eventually develop diabetes mellitus [8,9].

In the Diabetes Prevention Program Outcomes Study, a random assignment was done for 20 years. Among 1,614 participants, 24% developed type 2 diabetes mellitus, and 14% (885) who had not developed diabetes were found to have DR. After complete analyses from the entire study duration of follow-up, as compared with the non-Hispanic White race, the American Indian race showed less association with DR, higher HbA1c, fasting, and two-hour plasma glucose levels during an OGTT, weight, and history of hypertension, dyslipidemia, and smoking were associated with DR more frequently [10].

According to the DETECT-2 study, DR was associated with an FPG of 6.5 mmol/l [10]. It also concluded that end-organ damage like DR and nephropathy can occur before the onset of type 2 diabetes mellitus [9]. It was observed that people with prediabetic conditions have an increased prevalence of microvascular disease, a greater rate of mortality, and a double coronary heart disease mortality rate as compared to people with normal glucose tolerance (NGT) [11,12]. It is seen that people with a prediabetic state and associated microvascular disease have a higher tendency to develop type 2 diabetes mellitus [13-16]. Throughout the world, DR prevalence in the diabetic population in the form of diabetic macular edema, proliferative DR, and sight-threatening DR including intragel hemorrhage and tractional retinal detachment is 7.0%, 34.6%, 6.8%, and 10.2%, respectively [17,18].

This is the reason why it is important to diagnose it early to save sight [9,19]. Nowadays, a commonly used method to screen the diabetic population for retinopathy is digital retinal photography, though it can vary from place to place [20]. Isolated retinopathies without diabetes and hypertension were found in the 2.6-8.6% range, which can be considered a prediabetic state [21,22]. The present study aimed to estimate and discuss the DR prevalence in the prediabetic population by conducting a systematic review of published literature using various databases as evidence and using the Preferred Reporting Items for Systematic Reviews and Meta-Analyses (PRISMA) guidelines.

## Review

Methods

Literature searches were done using the Web of Science, CINAHL, Google Scholar, Cochrane, EMBASE, and PubMed databases from inception to April 2023. Our search included the words prediabetes, DR, and risk factors. All searches were looked at for methodological quality and evidence.

Inclusion and Exclusion Criteria for Literature Search

The inclusion and exclusion criteria for the literature search encompassed various facets. These comprised study types, sampling methods, and specifically targeting the prediabetic population. The studies included those reporting the prevalence of conditions such as DR, vision-threatening DR, or clinically significant macular edema among individuals with prediabetes or IGT. Clear definitions of prediabetic states were delineated, considering specific fasting and post-glucose ingestion plasma glucose levels. These criteria, aligned with ADA guidelines, specified IFG and IGT thresholds. Additionally, HbA1c levels between 5.7% and 6.4% were incorporated to define the prediabetic state as per ADA guidelines. DR was characterized by retinal abnormalities observed through various diagnostic methods. Exclusion criteria involved studies conducted solely in clinical settings or hospitals, clinical trials, and duplicates, along with those lacking full-text articles.

Outcomes

Outcomes were defined as primary and secondary. The primary outcome included the evident retinal findings on fundus photographs that comprised the DR in the prediabetic population, as defined by the International Clinical Diabetic Retinopathy Severity Scale (ICDRSS) classification [24]. Features observed on retinal photography were (1) microaneurysms, (2) dot and blot hemorrhage, (3) hard exudates, (4) cotton-wool spots, (5) venous beading and looping, (6) intraretinal microvascular abnormalities, (7) new vessels at the optic disc or elsewhere, and (8) intragel or pre-retinal hemorrhage. Some studies reported additional data on imaging with fundus fluorescein angiography (FFA) and optical coherence tomography (OCT). Additional data such as weight, BMI, blood pressure, urine protein, etc. were also studied for diagnosing prediabetes and cardiovascular and metabolic syndrome [1-3,25,26].

Study Selection and Data Collection

**Figure 1:**
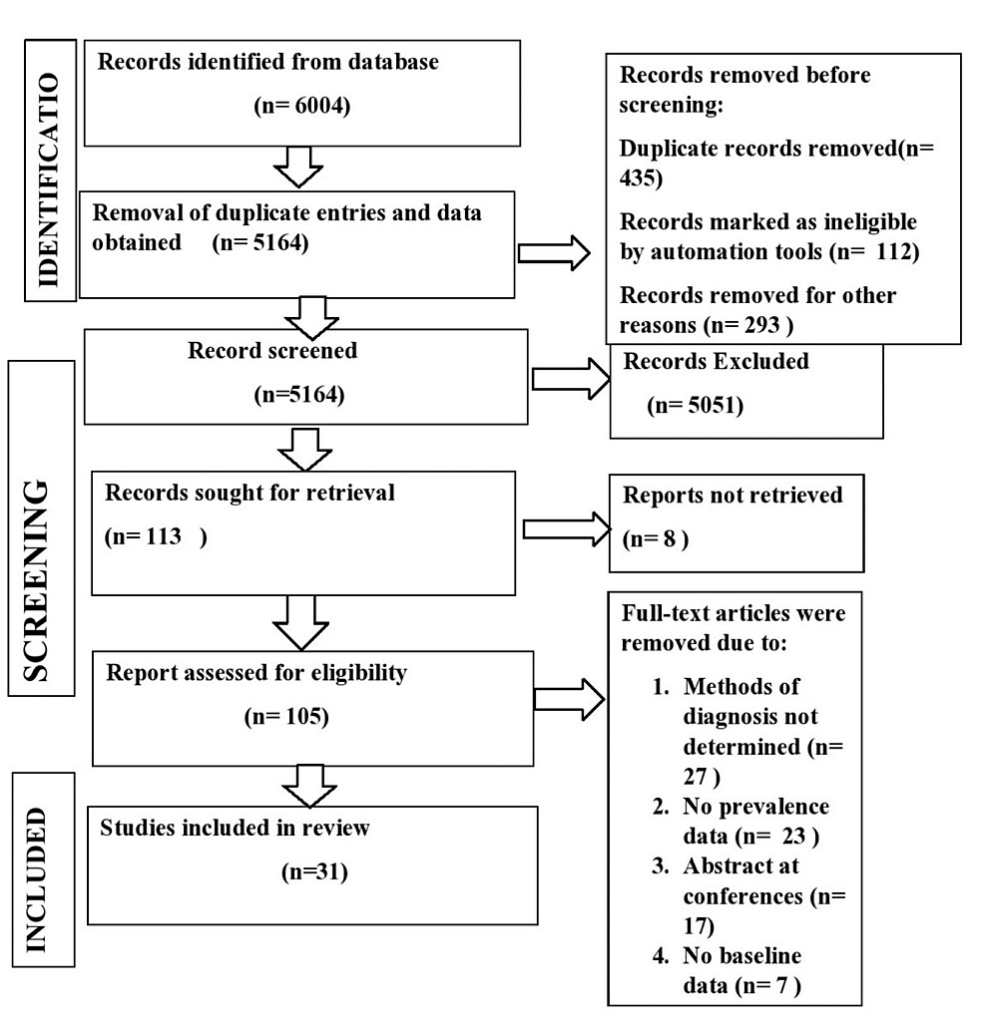
PRISMA flowchart showing the study selection process

One of the reviewers (MS) screened titles and abstracts to see if the study matched the eligibility criteria or not independently. Disagreements on the studies were resolved by discussion. Articles fulfilling the criteria were selected for full-text assessment. All population-based studies were considered, and only prediabetic populations were considered in the whole study, where different groups were a part of the study. PRISMA guidelines were followed, and the study process is shown in Figure 1. The prevalence was also estimated for comorbid ocular conditions like cataracts and cardiovascular risk factors, e.g., raised blood pressure and metabolic syndrome.

Results 

Characteristics of the Studies

From electronic database searches, all the duplicate entries were removed, 105 full-text articles were selected, and individuals (10,539) with prediabetes were included for final evaluation. Thirty-one studies were included after the screening. Population-based data were used in 26 studies (83.87%). The prediabetic population screened was 10,539. The estimated prevalence of retinopathy ranged between 0.3% and 20.9%, showing a median prevalence of 8.1%. The interquartile range (IQR) is 4.2-11%, with high variation in rates due to different screening methods used to diagnose and grading methods used for retinopathy and study populations. Several studies comparing the population with normal GTT with impaired GTT inferred that there was a lower prevalence of retinopathy in the normal GTT population (3.0%, IQR 0.3-7.4%). The estimated DR prevalence in prediabetes was found to be 6.7% (IQR 1.9-10.1%) which is higher than normal GTT. According to this data, a greater DR prevalence was found in the prediabetic population than in the previous systematic reviews. Of the prediabetic population studied, 27 studies reported age data. Male predominance was observed among the 31 studies that reported gender data, wherein gender ratios ranged from 17.7% to 69.2% male. Details of the studies included in the review are shown in Table 1.

**Table 1:** Characteristics of the studies included in the review - data not reported, * prediabetes group defined by HbA1c criteria only, ^$^ average value including other study groups (NGT, type 2 diabetes mellitus) ChS: cohort study, CSS: cross-sectional study, HBS: hospital-based study, RCT: randomized-controlled trial, PBS: population-based study, IFG: impaired fasting glucose, IGT: impaired glucose tolerance, NGT: normal glucose tolerance, HAm: Hispanic, NHBp: non-Hispanic Black, NHWp: non-Hispanic White

Author	Country	Study period	Design	Study group(s)	Sample size (prediabetics)	Mean age ± SD or median age (range)	Male gender (%)	Race or ethnicity
White et al. (2022) [10]	USA	16 years up to 2020	PBS, ChS	IGT	747	10.39	29.85	NHW, American Indian
Nagi et al. (1997) [27]	USA	1982-1990	PBS, CSS	IGT	288^$ ^(incl NGT)	45^$^ (15-93 incl NGT)	51^$^ (incl NGT)	Pima Indians
Penman et al. (2015) [28]	USA	2009-2012	HBS, CSS	IGT	266	65.7±9.6	40.2	African American
Tyrberg et al. (2008) [29]	Sweden	-	RCT	IFG	154	-	58.4	-
Hanssen et al. (2020) [31]	Netherland	2010-2013	PBS, ChS	IFG or IGT	478	61.6±7.6	54	-
Guney et al. (2022) [32]	Turkey	Jan 20219 to Sep 2019	PBS, CSS	IFG or IGT or combined IFG and IGT	86	-	-	Turkish
Chen et al. (2012) [33]	China	2005-2007	PBS, CSS	IGT	110	50.33±12.2	40.9	Chinese
Gabriel et al. (2020) [34]	Australia, Bulgaria, Austria, Kuwait, Poland, Serbia, Spain, Turkey	Ongoing	RCT	IFG or IGT	809	58.5±7.6	41.9	
Dowse et al. (1998) [35]	Mauritius	1987-1992	PBS, CSS	IGT	165	-	45.2^$^	Indians, Creoles, Chinese
Klein et al. (1991) [36]	USA	1984-1987	PBS, CSS	IGT	418	-	39	White
Collins et al. (1995) [37]	Samoa	1978-1991	PBS, CSS	IGT	97	-	37.2	Samoan
Rajalakshmi et al. (2022) [38]	India	2019	PBS, CSS	IFG or IGT or IFG with IGT	192	48±13	55.2	Indian
van Leiden et al. (2002) [39]	Netherland	1989-1892	PBS, CSS	IGT	177	64.2±7.3	51	Caucasians
Sundling et al. (2012) [40]	Norway	2004-2005	PBS, CSS	IGT	38	57±15	36.8	-
Lamparter et al. (2014) [41]	Germany	2007-2008	PBS, ChS	PD*	1112	59.9±9.1	51.8	-
Kawasaki et al. (2006) [42]	Japan	2000-2002	PBS, CSS	IGT	303^$ ^	58.6^$^	42.3^$^	Japanese
Arshid et al. (2022) [43]	India	2020 to 2022 (2 years)	HBS, CSS	IFG or IGT	200	56.7	53.5	Indian
Pang et al. (2011) [44]	China	1996-2007	PBS, CSS	IGT	865	62.3±10.8	42.9	Chinese
Bower et al. (2013) [45]	USA	2005-2008	PBS, CSS	PD*	636	-	-	NHWp 41%, NHBp 30%, HAm 29%
Bhargava et al. (2014) [46]	Singapore	-	PBS, CSS	PD*	829	-	-	Indian
Munch et al. (2011) [48]	Denmark	1991-2001	PBS, CSS	IFG or IGT or IFG with IGT	275	-	-	-
Callaghan et al. (2020) [49]	USA	2015	HBS, CSS	IFG or IGT	56	44.7±11.4	17.9	White 78.8%, White 69.6%, White 87.8%
Hu et al. (2015) [50]	China	2006	PBS, CSS	IFG or IGT	657	45.6±1.3	35	Chinese
Tikellis et al. (2007) [51]	Australia	1999-2000	PBS, CSS	IFG or IGT	960	58.9±13.5	43	-
Hermans et al. (1998) [52]	Egypt	-	PBS, CSS	IGT	103	-	41	-
Dyck et al. (2012) [53]	USA	2000-2005	PBS, CSS	IFG or IGT or IFG with IGT	174	64 (22-76)	67.8	-
Sokolowska et al. (2016) [54]	Poland	-	HBS, CSS	IFG or IGT	61	58	33.3	
Akhter et al. (2013) [55]	Bangladesh	-	PBS, CSS	IFG or IGT	54	-	56	Bangladeshi
Rajala et al. (1998) [56]	Finland	1990-1992	PBS, CSS	IGT	204	-	43.9^$^	-
Kumar et al. (2021) [57]	India	Feb 2019 to Aug 2020	HBS, CSS	IFG or IGT	100	55.5±8.7	58	Indian
Shrestha et al. (2023) [58]	Nepal	Jan 2022 to April 2022	PBS, CSS	IFG or IGT	141	60.69±13.1	-	Nepalis

Studies were conducted in 26 different countries. Out of 31, eight studies were from the European continent, seven were from the United States of America (USA), 13 studies were from the Western Pacific (China, Japan, Singapore, India, Turkey, Bangladesh, Australia, Nepal, and Samoa), and one each in Africa (Mauritius) and the Eastern Mediterranean (Egypt). One of the studies included a total of nine countries [27]. Among the studies, 20 reported data on race or ethnicity, and three examined Pima Indians, African Americans, and non-White Hispanics with American Indians, all of whom are from the USA exclusively [10,27,28]. The majority of published data were from cross-sectional and population-based studies (28/31, 90.32%).

There was a wide range of sample sizes, varying between 38 and 1112. Twenty-two studies defined prediabetes according to WHO criteria and eight according to ADA criteria. Among them, two studies used non-standard WHO criteria, one could not define prediabetes, and one used non-standard IFG criteria (Table 2) [29].

**Table 2:** Prevalence of DR in the prediabetic population - data not reported, IFG: impaired fasting glucose, IGT: impaired glucose tolerance, NGT: normal glucose tolerance, PDP: prediabetic population, ADA: American Diabetes Association, CSMO: clinically significant macular oedema, DRDSS: Diabetic Retinopathy Disease Severity Scale, ICDRSS: International Clinical Diabetic Retinopathy Severity Scale, ETDRS: Early Treatment Diabetic Retinopathy Study, HE: hard exudate, PDR: proliferative diabetic retinopathy, WES-DR: Wisconsin Epidemiologic Study of Diabetic Retinopathy, WHO: World Health Organization, FFA: fundus fluorescein angiography

Author	Study group(s)	Sample size	Definition of prediabetes	Definition of retinopathy	Method of diagnosis of retinopathy	No. of retinopathy (n )	Prevalence of retinopathy (%)	Secondary outcome
White et al. (2022) [10]	IGT	747	WHO	ETDRS	Stereoscopic fundus photography	127	15	PDR: 0%
Nagi et al. (1997) [27]	IGT	288 (incl NGT)	WHO	Modified Airlie-House	Mydriatic, imaging- 2-field, 45-degree digital	8	12	-
Penman et al. (2015) [28]	IGT	266	WHO	ETDRS ≥14	Mydriatic, 7-field color digital	25	9.4	CSMO: 7.8%
Tyrberg et al. (2008) [29]	IFG	154	Nonstandard	Alternative WES-DR ≥21	Mydriatic, 2-field, color film	16	10.4	-
Hanssen et al. (2020) [31]	IFG or IGT	478	WHO	ETDRS and ICDRSS	Color digital	-	0.3	-
Guney et al. (2022) [32]	IFG or IGT or combined IFG and IGT	86	WHO	ETDRS	on FFA findings	16	18.6	PDR: 0%
Chen et al. (2012) [33]	IGT	110	ADA	DRDSS	FFA using TOPCON TRC 50IX	23	20.9	PDR: 0%
Gabriel et al. (2020) [34]	IFG or IGT	809	WHO	ETDRS ≥14	Mydriatic, 3-field color digital	34	4.2	-
Dowse et al. (1998) [35]	IGT	165	WHO	Airlie-House	3-field, 45-degree digital	15	9.1	PDR: 0%
Klein et al. (1991) [36]	IGT	418	WHO	≥15	Dark-adapted, 1-field, 45-degree	6	1.4	-
Collins et al. (1995) [37]	IGT	97	WHO	Airlie-House	Mydriatic, 3-field, 45-degree	7	7.2	PDR:1%
Rajalakshmi et al. (2022) [38]	IFG or IGT or IGT with IFG	192	WHO	ICDR	Nonmydriatic ultrawide field fundus photography	12	6.3	PDR: 0%
Rajalakshmi et al. (2022) [38]	IGT	177	WHO	EURODIAB: ≥1 MA, Hg or hard exudate	Mydriatic, 2-field color digital	-	11	HE: 6%
Sundling et al. (2012) [40]	IGT	38	WHO	DRDSS: 5 stages	Nonmydriatic, imaging-1-field, 45-degree color digital	1	2.9	-
Lamparter et al. (2014) [41]	PD*	1112	ADA	ETDRS	Dark-adapted, 2-field	75	8.1	CSMO: 0.2%
Kawasaki et al. (2006) [42]	IGT	303	WHO	No standard, any MA, Hg, or exudate	Nonmydriatic, 1-field, 45-degree color digital	-	14.1	-
Arshid et al. (2022) [43]	IFG or IGT	200	WHO	ETDRS	-	12	6	PDR: 0%
Pang et al. (2011) [44]	IGT	865	ADA	DRDSS: G1-4	Dark-adapted, imaging-1-field, 45-degree color digital	22	2.5	CSMO: 2.4%
Bower et al. (2013) [45]	PD*	636	WHO	ETDRS ≥14	Nonmydriatic, 2-field, 45-degree color digital	-	9.7	-
Bhargava et al. (2014) [46]	PD*	829	ADA	ETDRS >14	Mydriatic, 2-field	55	6.6	-
Munch et al. (2011) [48]	IFG or IGT or combined IFG and IGT	58, 152.5, 64.6	WHO	ETDRS ≥15	Mydriatic 7 field 60 deg color digital	-	11.7 8.4 9.5	-
Callaghan et al. (2020) [49]	IFG or IGT	56	ADA	-	Nonmydriatic color digital	-	1.9	-
Hu et al. (2015) [50]	IFG or IGT	657	ADA	ETDRS	Dark-adapted, 1-field	9	1.4	-
Tikellis et al. (2007) [51]	IFG or IGT	960	WHO	Wisconsin	Nonmydriatic, 2-field, 45-degree color digital	66	6.9	-
Hermans et al. (1998) [52]	IGT	103	WHO	Wisconsin	Mydriatic digital imaging	-	1.9	-
Dyck et al. (2012) [53]	IFG or IGT or IFG with IGT	118, 19, 37	ADA and WHO	NSC: R0-R3	7-field color digital fundus imaging	-	IFG-4.3, IGT-9.9, IFG with IGT-8.7	WHO cut-off for IFG (6.1 mmol/L) but also included abnormal A1c as per ADA
Sokolowska et al. (2016) [54]	IFG or IGT	61	-	-	Color digital	6	9.8	-
Akhter et al. (2013) [55]	IFG or IGT	54	WHO	ETDRS	Mydriatic, 3-field color	7	13	-
Rajala et al. (1998) [56]	IGT	207	WHO	University Hospital of Oulu classification: G1-4	Dark-adapted, 1-field, 45-degree film	-	2	-
Kumar et al. (2021) [57]	IFG or IGT	100	ADA	ETDRS	-	14	14	PDR: 0%
Shrestha et al. (2023) [58	IFG or IGT	141	WHO	ETDRS	Slitlamp biomicroscopy and indirect ophthalmoscopy	8	5.67	PDR: 0%

Risk of Bias Assessment

The risk of bias assessment was done by assessing individual items that are deemed to reflect the methodological risk of bias by using the modified McMaster Critical Appraisal Tool. Points were scored on individual items to be assessed for risk. The majority of studies (22/31, 70.96%) showed a "low" risk of DR, while the remaining nine showed a "moderate" risk. Most studies (10 studies) did not show proper randomization of the study population. Few studies lacked pharmacological mydriasis (14 studies), raising questions of validity and reliability. There was clinical and statistical heterogeneity (94%) and a lot of inequality in study populations, the degree of fields used to capture retinal photographs, the use of different retinopathy classifications, the use of mydriatic agents or nonmydriatic fundus pictures, and diagnostic criteria for prediabetes [30]. In studies where quantitative data availability was found, median prevalence and ranges were calculated. In the absence of quantitative data, we opted for a narrative analysis. Details are shown in Table 2.

Primary Outcome

**Figure 2:**
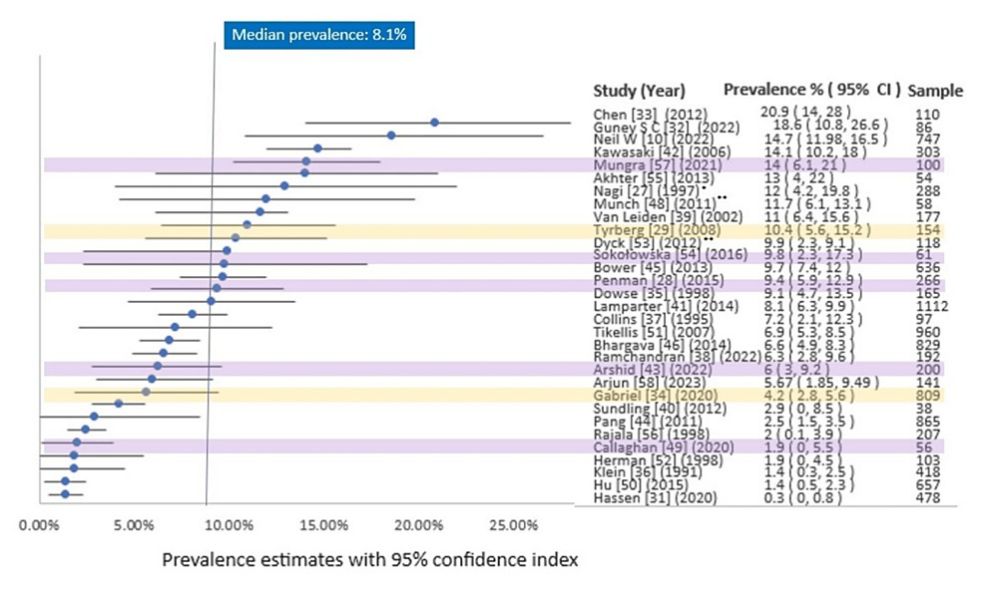
Forest plot of the prevalence of retinopathy in the prediabetic population from the included studies ∗ Prediabetes group size estimated from the reported retinopathy prevalence and the number of affected individuals. ∗∗ Aggregate prevalence estimates presented for impaired fasting glucose (IFG), impaired glucose tolerance (IGT), and combined IFG and IGT (IFG-IGT). All studies are population-based, except three hospital-based studies (lavender highlights) and two randomized-controlled trials (yellow highlights). CI: confidence interval

The estimated prevalence of retinopathy ranged between 0.3% and 20.9% (median 8.1%, IQR 4.2-11%) [31-33]. However, the estimation of prevalence varied widely. A study from the Netherlands showed 0.3% (n=478), whereas a study from Turkey showed a prevalence of 18.6% (n=86) [31,32], and Chen et al. showed 20.9% [33]. The median sample size for prediabetes was 165 (range 38-1112). The prevalence of DR in the studies included is shown in Table 2 and Figure 2.

Subgroup Analyses of Primary Outcome

Wherever we found a lack of quantitative data subgroups, a narrative analysis was done. The median estimated retinopathy was 8.1% (0.3-20.9%). In the USA, the value was 9.4% (1.4-15%), while the median value for Europe was 8.1% (0.3-14.0%) [31,34]. In Asia, the reported value was 6.3% (1.4-20.9%). Only a study from the African continent showed 9.1% [35]. According to the data available, the USA (10.28%) estimated the mean highest prevalence of DR in prediabetics, followed by Asia (9.6%) (Table 2).

Demographic Features

Only three of the studies had age-specific data on the prevalence of prediabetics. Most of the studies found that the maximum number of participants in the study fell between 50 and 70 years of age. Klein et al. and Herman et al. observed a higher DR prevalence in people aged more than 50 years compared to those aged below 45 [36,52]. All studies showed a higher prevalence in males [18]. A higher prevalence of retinopathy was found in females only in one study [36]. In two of the studies, female participants were more frequent [10,34]. Two studies compared ethnicity with respect to DR prevalence, with a higher prevalence in Hispanic White people (10.0%) and non-Hispanic Black people (11.6%) [35,10]. The highest prevalence of DR (20.9%) was found in Turkey and China [32,33].

Prevalence of DR in Subtypes of Prediabetes

A median prevalence of 8.5% (1.9-18.6%) for IGT was reported by 15 studies, and a median DR prevalence in individuals with IFG was 10.4% (4.3-15%) concluded in five studies. Among the studies, one study opted for the upper limit of IFG as <6.1 mmol/l, which differed from ADA and WHO criteria (<7.0 mmol/l). Four studies observed DR prevalence combined with IFG/IGT, with a median prevalence of 8.5% (0.3-15%) for DR in prediabetics. Prevalence estimates for retinopathy among participants with IFG or IGT, with a median prevalence of 6.8% (1.4-18.6%), were reported by 12 studies (Table 2).

Grade of Retinopathy

Different studies used different types of classification to grade retinopathy. Some studies utilized the Early Treatment Diabetic Retinopathy Study (ETDRS) classification, while others employed the international classification of DR. Penman et al. reported the prevalence of retinopathy by ETDRS grade [28], involving 266 participants with IGT. The ETDRS grades revealed 15, 20, and 35 grades of retinopathy, with frequencies of 12 (4.5%), 9 (3.4%), and 4 (1.5%), respectively. In a study conducted by Collins et al. (n=97), a single case of advanced DR was reported in a Samoan population [37]; however, a study conducted by Dowse et al. (n=165) did not find a single case of proliferative DR where a mixed population of Chinese, Indian, and Creole were participants [35]. Rajalakshmi et al. reported DR in 12 (6.3%) participants with nine (4.7%) mild non-proliferative DR (NPDR) and three (1.6%) with moderate NPDR. None reported severe, sight-threatening DR [38].

Prediabetics and Comorbid Ocular Pathology

Clinically significant macular edema was reported in very few studies. A 6% DR prevalence was reported by van Leiden et al. (n=165), which included only NPDR cases among participants with IGT [39]. In a study by Sundling et al. (n=38), one participant (2.6%) reported having hypertensive retinopathy among individuals with IGT [40]. In IGT, NGT, and diabetic populations, there was no statistical difference in the prevalence rate of age-related macular degeneration or glaucoma. Cataract and pseudophakia status were hardly reported; hence, the prevalence of cataracts could not be measured reliably in prediabetics [41].

Comorbid Risk Factors

The included studies showed an association with hypertension, showing a prevalence of 61% (31-71%) for hypertension in prediabetes. In one study, microalbuminuria was found in 11% of prediabetics, which is a sign of nephropathy [42-44].

Diagnosis of Prediabetes

Twenty-two studies showed median prevalence that used WHO criteria (9.6%, 0.3-18.6%), whereas eight studies that used ADA criteria showed prevalence estimates (2.5%, 1.4-8.6%). HbA1c-related data were available in 14 studies, whereas data from 14 studies was available for FPG and 15 studies for OGTT. Three of the studies only used HbA1c as criteria for the diagnosis of prediabetes, wherein the median prevalence was 8.1% (6.6-9.7%) (Table 2) [41,45,46].

Methods Used to Diagnose DR

This data, collected from multiple studies, is based on retinal images captured using various degrees of field, ranging from one- to seven-field fundus imaging. In studies conducted using mydriatic drugs, the highest median DR prevalence (10.9%, 1.9-20%) was found. Studies performed without mydriatic drops showed a prevalence of 6.3% (1.9-14%). Five studies that had done imaging after dark adaptation obtained physiological mydriasis and observed a lower prevalence rate of 2.0% (1.4-8.1%). The rest of the study didn’t provide sufficient information.

Secondary Outcomes

In the current review, no study showed any microvascular abnormalities that were not defined as primary outcomes (features of DR). Only three studies observed the presence of maculopathy and its prevalence in the prediabetic population. The prevalence rate of CSMO was found to be 0.2% and 2.4% by Lamparter et al. (n=922) and Pang et al. (n=865), respectively [41,44,47]. Most of the studies have no maculopathy, and retinopathy was restricted to a mild form only [28,38].

Multivariate analysis

**Figure 3:**
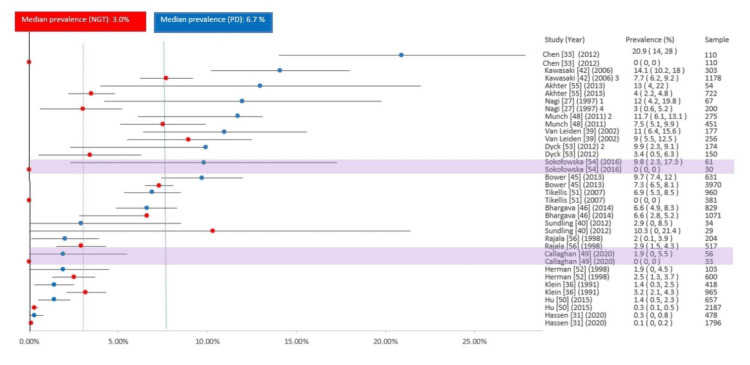
Forest plot of the prevalence of retinopathy in the prediabetic population and NGT from included studies reporting data for both groups Normal glucose tolerance (NGT) prevalence estimates in red and prediabetes prevalence estimates in blue. ^1^ Prediabetes group size estimated from reported retinopathy prevalence and number of affected individuals. ^2^ Impaired fasting glucose (IFG), impaired glucose tolerance (IGT), and combined IFG and IGT (IFG/IGT) retinopathy prevalence estimates aggregated with total prediabetes group size used for 95% confidence interval (CI) estimation. ^3^ NGT group size estimated from the total study sample minus the reported prediabetes population. ^4^ Prediabetes group size estimated from reported retinopathy prevalence and number of affected individuals. All studies are population-based, except two hospital-based studies (lavender highlights).

Tables 1-2 show the summary of all the study groups. Exploratory post hoc comparisons were performed to observe if the DR prevalence in the prediabetic population is greater than that of NGT. Among the 31 studies, 18 also reported the DR prevalence in NGT. In this review of the included studies, the median observed DR prevalence in prediabetic populations was 8.1% (IQR 4.2-12%). The prevalence of NGT observed was 3.0% (IQR 0.3-7.4%), which is summarized in Figure 3. There was a wide variation in DR prevalence and sample sizes in NGT, ranging from 0.1% to 10.3% and 29 to 3970 participants, respectively. In this review, 14 studies out of 18 (77.7%) observed a higher DR prevalence rate in prediabetic populations than NGT.

Discussion

We observed the median DR prevalence in the prediabetic population to be 8.1% (range 0.3-20.9%) in this systematic review, of which 22 studies (70.96%) reported a prevalence of ≥5% in the prediabetic population, which is higher than the review done by Kirthi et al. [16]. This may be due to recent Asian studies, which observed a higher prevalence rate [32,39]. However, the range was wide from 0.3% to 20.9%. This might be due to physiological and pharmacological mydriasis as well as the differences in the field observed in different studies. Despite variations, multivariate analysis revealed a higher median prevalence of DR (6.7%) in prediabetes within the same studies compared to NGT (3.0%). There was a low prevalence of moderate retinopathy and CSMO in prediabetic populations, which may be due to insufficient information on the grade of retinopathy.

Demographic Risk Factors

Important risk factors for DR include ethnicity, age, disease duration, and hyperglycemia status [17]. The mean ages of prediabetic participants were matched to those reported in diabetic population studies. Only two studies reported a higher rate of DR prevalence in females, despite reports showing higher retinopathy prevalence in males in diabetes or prediabetes conditions [10,23,47]. One study reported data on ethnicity and observed an excess DR prevalence among non-Hispanic Black individuals, similar to the diabetic population [17], and others compared non-Hispanic Whites with American Indians.

HbA1c, IFG, and IGT Comparisons

Retinopathy prevalence rates were observed differently for participants with IFG, IGT, and combined IFG and IGT subgroups, of which four studies published separate data for all subgroups, though many had combined IFG and IGT participants [38]. Individuals where combined IFG and IGT data were available were shown to have a higher risk of conversion p to type 2 diabetes mellitus (15-19%) as compared to only IFG (6-9%) or IGT (4-6%). However, in view of DR, things are not similar [59]. Most of the studies showed more DR prevalence in IGT than in the IFG subgroup [59]. Pathological mechanisms varied in IFG and IGT based on the insulin resistance that originated, which was predominantly hepatic and muscular, respectively [8,9]. This explanation justifies the variations in retinopathy prevalence in prediabetics. HbA1c is an indicator of chronic glycemia, whereas the OGTT is a single-time point measurement. DETECT-2 demonstrated a relationship between HbA1c and OGTT similar to OGTT [60-63]. Single HbA1c, when used as a diagnostic criterion (5.7-6.4%) showed similar annualized incidence rates of diabetes as in IFG and IGT participants (7%) [9].

The Severity of Retinopathy

Early-stage retinopathy was found predominantly using ETDRS grades, and microaneurysms were the most common lesion observed [61]. Among the studies, only two with 262 individuals observed the prevalence of advanced DR in the prediabetic population [62]. In a study conducted by Rajalakshmi et al., the DR prevalence was 6.3% (n=12). Nine (4.7%) individuals reported mild NPDR and three (1.6%) had moderate NPDR. None of them had severe DR [38].

Methods of Retinal Imaging

Pharmacological mydriasis gives more field visibility for DR screening than using non-mydriatic fundus cameras (3.7% compared to 19.7%, respectively) [63]. Our review showed greater median DR prevalence in studies wherein mydriatic agents were used compared to studies with no dilatation or physiological dilatation of the pupil. The extent of retinal fields for imaging used was also different among studies, which may have reflected on DR prevalence [64]. The correlation of seven-field fundus imaging was well observed with a clinical examination done by an ophthalmologist [65].

Comorbid Ocular and Metabolic Disease

Limited data is available on ocular and metabolic disorders. Insufficient data on the lens status was published (phakic or pseudo-phakic). Very few studies mentioned the cataract status [38]. Studies showed that in prediabetes hypertension, components of metabolic syndrome like dyslipidemia and BMI were higher compared to NGT. Also, serum uric acid levels, estimated glomerular filtration rate (eGFR), and antihyperlipidemic drugs were associated with DR [32]. Control of blood glucose levels and retinal damage are well correlated. Hypertension was found to be one of the important risk factors for retinopathy [66-68]. Various studies in human and animal models postulated that retinal vascular endothelial dysfunction and chronic inflammatory processes are common etiopathogeneses for both hypertensive and DR [21,69]. In prediabetes, it was observed that there was low-density lipoprotein and raised triglycerides, leading to dyslipidemia [70,71]. Several studies have reported associations between microalbuminuria and DR in prediabetics [57,72]. The association of retinopathy with dyslipidemia is varied, and there are reports published on the associations of hypercholesterolemia and retinal lesions (hard exudates) and also between hypertriglyceridemia- and diabetes-induced retinopathy [33,58,73,74].

Limitations of the current review

Among 31 studies, only eight (25.8%) included participants above 500, and six studies (19.3%) had participants less than 100. Studies with fewer participants carry a risk of bias in estimating the prevalence and have less reliability. Diagnostic criteria for prediabetes differed among the studies, and data on IFG, IGT, and IFG and IGT of the same individuals were reported by only two studies. There was higher clinical heterogeneity in diagnostic criteria and statistical variabilities found in study design and methods, so a meta-analysis was limited.

## Conclusions

DR prevalence was found to be higher in the prediabetic population (median 8.1%) as compared to the non-diabetic population. This review indicates a sizable number of people with prediabetes have end organ damage that is below the threshold level of glycemia for diabetes. There is an increase in the conversion rate of prediabetes to diabetes by around 10% annually, and this review shows evident early involvement of organs like the eye and renal resulting in DR and nephropathy, respectively, which needs greater monitoring to avoid end-organ damage in prediabetes. Risk factors like hypertension and components of metabolic syndrome like dyslipidemia and body BMI were higher in prediabetics compared to NGT. Also, serum uric acid levels, eGFR, and antihyperlipidemic drugs were associated with DR. Control of blood glucose levels and retinal damage are well correlated. Hypertension was found to be one of the important risk factors for retinopathy, along with prediabetes. Monitoring of these associated risk factors is also important.
